# Study on the role of transcription factor SPI1 in the development of glioma

**DOI:** 10.1186/s41016-022-00276-2

**Published:** 2022-04-01

**Authors:** Baoshun Du, Wuji Gao, Yu Qin, Jiateng Zhong, Zheying Zhang

**Affiliations:** 1 Second Department of Neurosurgery, Xinxiang Central Hospital, Xinxiang, Henan 453003 P.R. China; 2 Department of Pathology, Xinxiang Medical University, 601 Jinsui Avenue, Xinxiang, Henan 453003 P.R. China

**Keywords:** Glioma, SPI1, PAICS, proliferation, migration

## Abstract

**Background:**

Glioma is a common malignant brain tumor. The purpose of this study was to investigate the role of the transcription factor SPI1 in glioma.

**Methods:**

SPI1 expression in glioma was identified using qRT-PCR and Western blotting. Cell proliferation was assessed using the CCK8 assay. Transwell and wound healing assays were utilized to evaluate cell migration. Additionally, cell cycle and apoptosis were detected using flow cytometry.

**Results:**

We observed that the expression level of SPI1 was up-regulated in glioma tissues, compared to normal tissues. Furthermore, we found that SPI1 is able to promote proliferation and migration of glioma cells *in vitro*. Flow cytometry results demonstrate that, compared to si-NC cells, si-SPI1 cells stagnated in the G1 phase, and down-regulation of SPI1 expression is able to increase rates of apoptosis. Double luciferase activity and chromatin immunoprecipitation assay results indicated that SPI1 can bind to the promoter sites and promote the proliferation and migration of glioma cells by regulating the expression of oncogenic PAICS.

**Conclusions:**

Our results suggest that SPI1 can promote proliferation and migration of glioma. Furthermore, SPI1 can be utilized as a potential diagnostic marker and therapeutic target for glioma.

**Supplementary Information:**

The online version contains supplementary material available at 10.1186/s41016-022-00276-2.

## Background

Glioma is a disease that seriously endangers human health. Thus far, the pathogenesis of glioma remains unclear. Clinically, patients suffer from the pain caused by radiotherapy and chemotherapy, which causes serious economic and mental burden to society and family.

The transcription factor SPI1 is an important regulatory protein that plays an extremely important role in blood cell differentiation, immune cell differentiation, as well as tumor development [1, 2]. To date, no studies have reported on the relationship between SPI1 Phosphoribosylaminoimidazole Carboxylase and Phosphoribosylaminoimidazolesuccinocarboxamide Synthase (PAICS) in glioma. Numerous studies demonstrated that PAICS can promote the proliferation and migration of many tumors [3–8]. Our previous study also found that PAICS plays an oncogene role in glioma [9]. In this project, we intend to further study the transcriptional regulation of SPI1 and PAICS through luciferase assay, gene transfection technology, Western blot and other techniques. Our goal was to explore the role of SPI1 in the occurrence and development of glioma with regards to regulation of PAICS, and provide a new direction for further exploration of glioma pathogenesis.

## Methods

### Clinical specimens and cell lines

Human gliomas and surrounding normal brain tissues requiring decompression were collected from the Xinxiang Central Hospital in Henan Province. Written informed consent was obtained from all patients or their relatives. All work was granted approval by the Medical Ethics Committee of Xinxiang Central Hospital. Tissue specimens were frozen in liquid nitrogen and stored at -80°C. The glioma cell line used in this study was obtained from ATCC, and cultured in RPMI 1640 (Hyclone) containing 10% Fetal Bovine Serum (FBS) (Gibco) at 37°C and 5% CO_2_.

### RNA extraction and qRT-PCR

RNA was extracted using the TRIZol reagent (Takara). The cDNA was synthesized using the Takara reverse transcription kit. Real-time quantitative RT-PCR experiments were performed using cDNA as the template. SYBR Green I (Takara) was added, and the primers were mixed. The mixture was run on Applied Biosystems 7500/7500 Fast (Applied Biosystems), with GAPDH as the internal control. All experiments were repeated three times. SPI1 reaction conditions were as follows: 95°C for 5 min, 40 cycles at 95°C for 1 min, Tm of 30 s, and then 72°C for 34 s. The ΔCt method was used to calculate relative expression of SPI1. The ΔCt values were used to compare the expression level of SPI1 in tumor vs the control group. ΔCt = Ct_SPI1_ – Ct_GAPDH_. The SPI1 primers are listed as follows. The forward primer was 5′- GCGACCATTACTGGGACTTCC - 3′ and the reverse primer was 5′- GGGTATCGAGGACGTGCAT -3′. GAPDH was used as an internal control. The primers for GAPDH were 5′- GACTCATGACCACAGTCCATGC - 3′ and 5′- AGAGGCAGGGATGATGTTCTG -3′.

### Construction of a SPI1-knockdown cell lines and transfection

The SPI1 siRNA was purchased from Gene Pharmaceutical (Shanghai, China). The nucleotide sequence for the SPI1 siRNA is 5'- GAGAGCTTCGCCGAGAACAACTTCA-3'. Transfection of siRNA oligonucleotides and plasmids was conducted with Lipofectamine 2000, which was purchased from Invitrogen, ThermoFisher Scientific (Catalog No.11668019, Massachusetts, USA).

### Transwell assays

Cultured cells in the logarithmic phase were digested by trypsin in order to make single cell suspension waves. The cell concentration was adjusted to (1-2)×l0^5^/mL. Next, 100 μl of the cell suspension was added to the upper chamber, and then 500 μl containing 10% of newborn bovine serum was added to the lower chamber. The cells were cultured for 48h at 37°C. The small chamber was taken out, fixed with 4% paraformaldehyde for 20 min, and stained with Giemsa for 15 min. Under the microscope, 5 fields were randomly chosen to count the number of cells that passed through the membrane in a 200X microscope. The influence of changes in SPI1 expression on cell migration was compared. The transwell chamber was purchased from Corning, Inc., USA.

### Wound healing assay

Cultured cells in the logarithmic state were seeded onto a 6-well plate with 8x10^5^ cells per well at 1-2 days before the experiment. The cells were incubated at 37°C in a 5% CO_2_ incubator. When the cells were grown to approximately 90% confluency, a 200 μl sterile spear was used to create a scratch on the 6-well plate. The liquid was then gently shaken and discarded to remove the floating cells. Pictures were taken under an inverted microscope at 10-times the field of view. Cell migration at the scratch was recorded at different time points.

### Cell cycle detection

The transfected cells were collected and centrifuged at 4°C. The supernatant was discarded, and fixed with pre-cooled 70% ethanol for 24 h. Then, the cells were washed with PBS. Next, 10 μl of RNase A solution was added to each sample, as well as 40 μl of PBS, according to the instructions of the cell cycle detection kit. The mixture was placed in the water bath at 37°C for 30 min. The red fluorescence at the excitation wavelength of 488nm was recorded by flow cytometry.

### Cell apoptosis detection

Cell apoptosis was evaluated by Annexin V/PI double labeling. The cells were digested and collected, washed twice with PBS, and made resuspended into a single cell suspension. Cell suspension was then incubated with Annexin V-FITC and PI at room temperature, and placed in the dark for 15 min. Flow cytometry was utilized to determine the results, and the CellQuest analysis software helped analyze the results.

### Double luciferase activity assay

The Dual-Luciferase ReporterAssay System (Catalog No. E1910) of Promega was utilized to quantify dual luciferase activity. Additionally, 48h after the plasmid was transfected, the medium was removed and washed twice with PBS. Next, 65 μl of 1× Passive Lysis Buffer (PLB) was added to each well. Then, the cell lysates were collected after gentle vibration at room temperature for 15 min. We utilized an automatic luminous detector, which required adding 20 μl of the cell lysate to each hole, and then adding 100 μl of LAR II to automatically detect the fluorescence value of firefly (F). After adding a 100 μl of the STOP&Glo@Reagent, the fluorescence value (R) was determined immediately. After reading the value, the data was saved for analysis of the detection results.

### Chromatin immunoprecipitation (ChIP)

ChIP assays were carried out according to the EZ-CHIP kit (Millipore, Temecula, CA, USA). The anti-SPI1 (Abclone) antibodies were utilized to precipitate the DNA-protein complex. The immunoprecipitated DNA was evaluated by PCR. Primers that were specific to the PAICS promoter containing E-box were 5’-TAAAGTTCATGGAAGCGAGGTG-3’ (forward) and 5’- TCACTCTGGGACTCGTGGG-3’ (reverse).

### Statistical analysis

Data was analyzed using the SPSS 20.0 statistical software. Quantitative data was presented as mean ± SD of at least three independent experiments. The differences between the independent experimental groups were assessed using a two-tailed Student's t-test. Differences were considered significant if p < 0.05*, p < 0.01**, p < 0.001***.

## Results

### Increasing SPI1 levels correlate with glioma progression

**Fig. 1.**
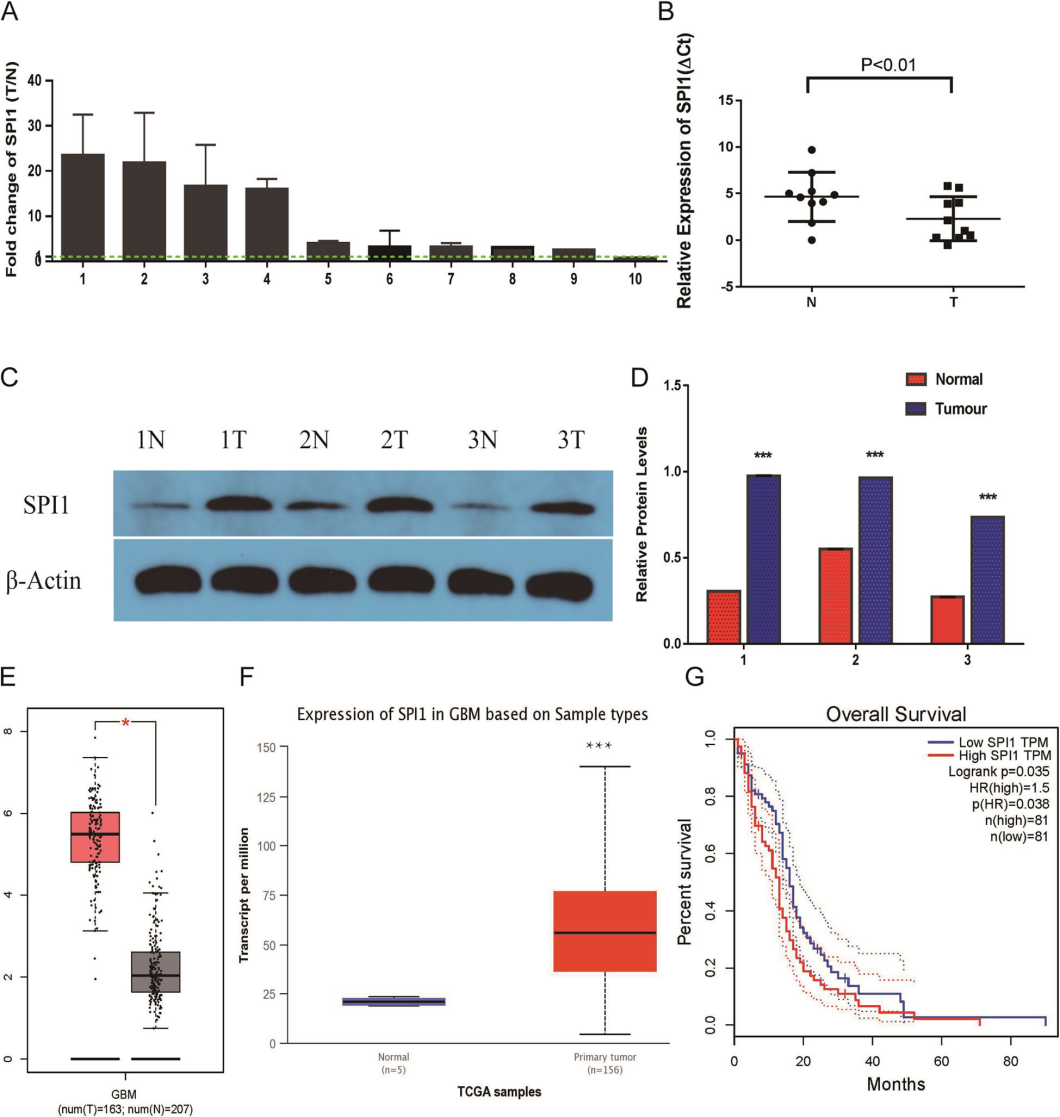
Expression levels of SPI1 in glioblastoma tissues. **a** RT-qPCR analysis of SPI1 expression in glioblastoma tissues compared with the control brain tissues. Error bars indicate the mean ± standard deviation of three independent experiments. (N: normal brain tissue, T: glioma tissue, green line represents T/N=1). **b** The value of ΔCt was used to show the expression level of SPI1 (ΔCt =Ct (SPI1) − Ct (GAPDH)) in the 10 paired human glioblastoma tissues and control brain tissues (*P* < 0.01). **c** Western blot analysis of SPI1 protein expression in three pair glioblastoma tissues compared with the normal tissues. The differences between tumor group and normal group were tested by using independent-samplest-test. **d** Protein expression level of SPI1 was normalized to GAPDH and quantified using Image J. Error bars represent the mean ± SD of 3 different tests. ***, *p* < 0.001. **e** Expression levels of SPI1 mRNA with glioma tissues and control tissues in GEPIA database . *, *p* < 0.05. **f** Expression levels of SPI1 protein with glioma tissues and control tissues in UALCAN database. **g** Survival curve of low SPI1 expression group and high SPI1 expression group

In order to investigate the role of SPI1 in glioma tumorigenesis, we determined the expression of SPI1 in 10 paired glioblastoma (GBM) tissues and adjacent normal counterparts by qRT-PCR. The expression of SPI1 was normalized to GAPDH, and results were presented as fold-change in tumor tissues relative to matched adjacent normal tissues. Formula =2^-ΔΔCt^ was utilized to calculate relative expression of SPI1 in tissues. Paired-samples t-test was used to determine the significance of the ΔCt values between the tumor and control group. The results revealed that SPI1 expression was increased in 9 of 10 GBM specimens (P < 0.05) (Fig. 1a, b). Additionally, western blot assay helped determine the protein expression of SPI1 in three paired GBM tissues. The results demonstrated that protein expression of SPI1 in GBM tissues was higher compared to paired adjacent normal tissues (P<0.05) (Fig. 1c, d). In the online GEPIA database (http://gepia.cancer-pku.cn/) and UACLAN database ((http://ualcan.path.uab.edu/), we analyzed the expression and survival of SPI1 [10, 11]. The results indicated that expression of SPI1 was significantly up-regulated in the tumor, and was associated with a lower survival rate in GBM (Fig. 1e-g). The protein expression of SPI1 in glioma tissues was validated through the use of data from Proteinatlas Version 19.3 (http://www.proteinatlas.org) (Supplementary Fig. S1) [12–14].

### SPI1 promotes glioma cell lines proliferation and migration *in vitro*

**Fig. 2.**
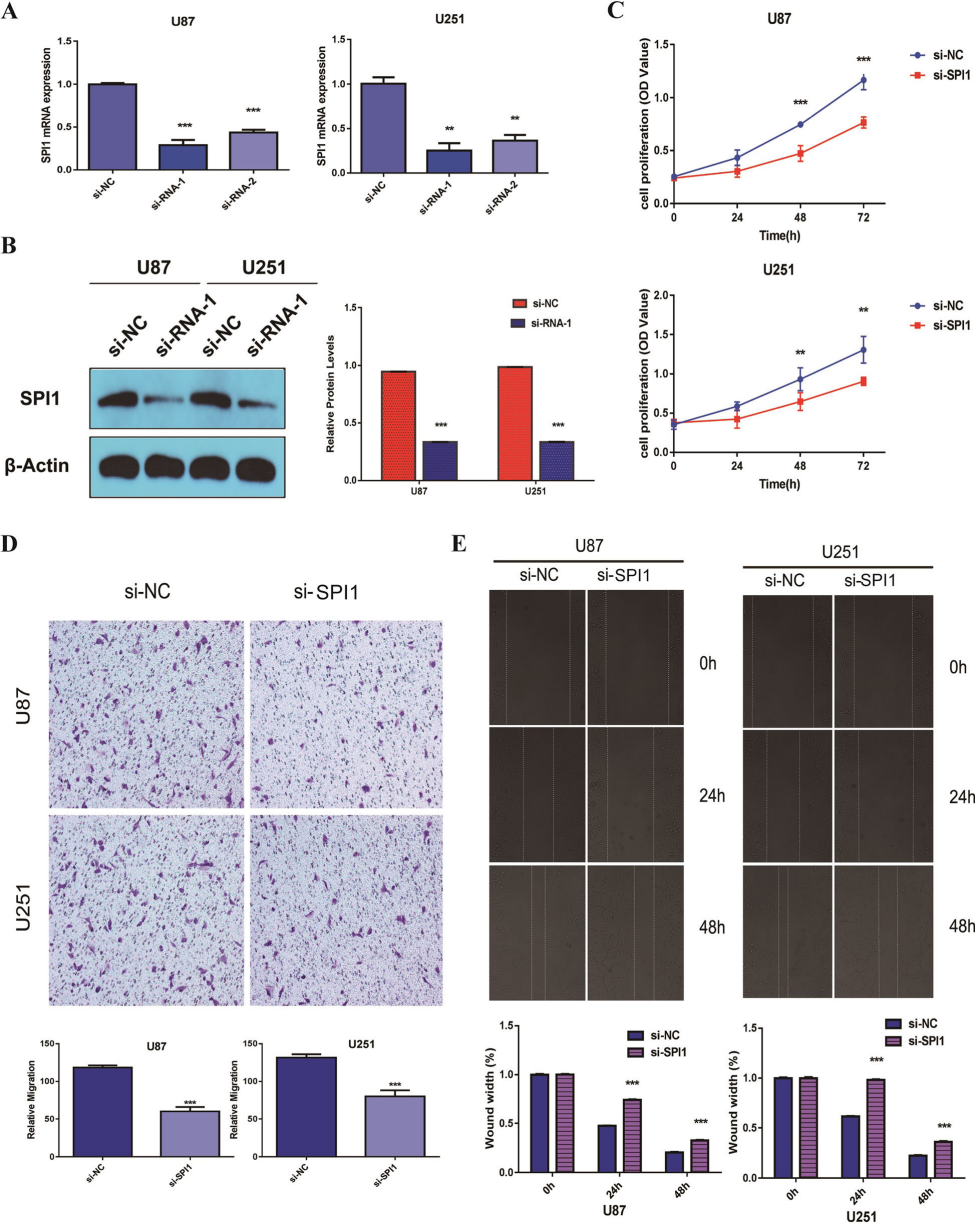
Inhibition of SPI1 in glioma cells weakened the cell growth and metastasis in vitro. **a** RT-qPCR analysis of SPI1 mRNA levels after the transfection of the si-NC or si-SPI1 small interfering RNAs. SPI1 expression was normalized to GAPDH, Error bars indicate mean ± SD of 3 independent experiments.**, *p*< 0.01; ***, *p*< 0.001. **b** Western blot analysis of SPI1 protein levels after the transfection of the si-NC or si-SPI1 small interfering RNAs. SPI1 expression was normalized to β-Actin, Error bars indicate mean ± SD of 3 independent experiments. ***, *p*< 0.001. **c** Knockdown of SPI1 inhibited cell proliferation on the basis of CCK8 assays. Error bars represent the mean ± SD of 5 independent experiments. **, *p*< 0.01;***, *p*< 0.001. **d** Inhibition of SPI1 decreased cell migration as determined by transwell assays. The bar chart represents the migration cell numbers. Error bars represent the mean ± SD of 5 different field. ***, *p*< 0.001. **e** Inhibition of SPI1 decreased cell migration as determined by wound healing assay. The bar chart represents the percentage of distance at 24h or 48h divided by the distance at 0h. Date are presented as mean ± SD of 3 independent experiments. ***, *p*< 0.001

In order to understand SPI1 functionality in glioma progression, we knocked down PAICS expression with siRNAs in the U87 and U251 cells, respectively. The qRT-PCR and western blot assays were performed to determine the expression of SPI1. The results demonstrated that the mRNA and protein expression of SPI1 was reduced (Fig. 2a, b). Furthermore, CCK8 results showed that the knockdown of SPI1 reduced cell growth (Fig. 2c). In addition, transwell and wound healing assay were used to detect motility, the results of which revealed that repression of SPI1 attenuates migration of U87 and U251 cells (Fig. 2d, e).

### Inhibition of SPI1 suppresses cell cycle and apoptosis in glioma cell lines

**Fig. 3.**
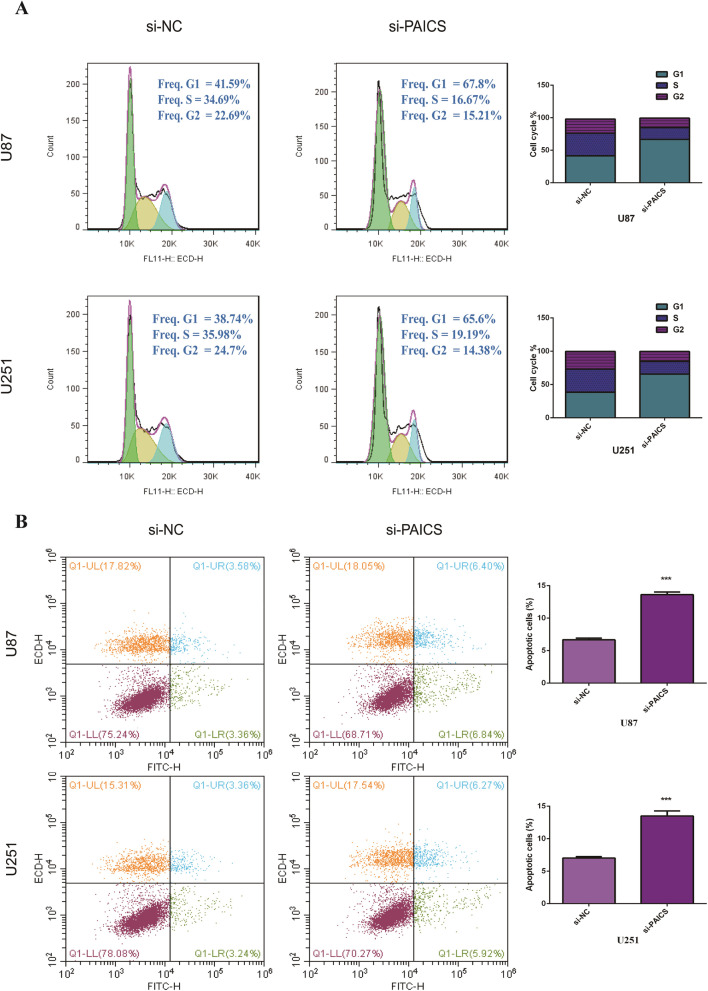
Knockdown of SPI1 in glioma cells inhibit cell cycle and promote apoptosis in vitro. **a** Cell cycle was analyzed by flow cytometry after knocking down PAICS. The results showed that PAICS depletion led G1 arrest. **b** Cell apoptosis was analyzed by flow cytometry after knocking down PAICS. The results showed that PAICS depletion leading to an increased rate of apoptosis

Flow cytometry revealed that knock down in the expression of SPI1 in U87 and U251 cells increased the proportion of cells in G0/G1 phase, and they decreased the proportion of glioma cells in the S phase (Fig. 3a). Furthermore, we discovered that a decrease in the expression of SPI1 increased the apoptotic rate of glioma cells (Fig. 3b).

### SPI1 is a transcriptional regulator of PAICS

**Fig. 4.**
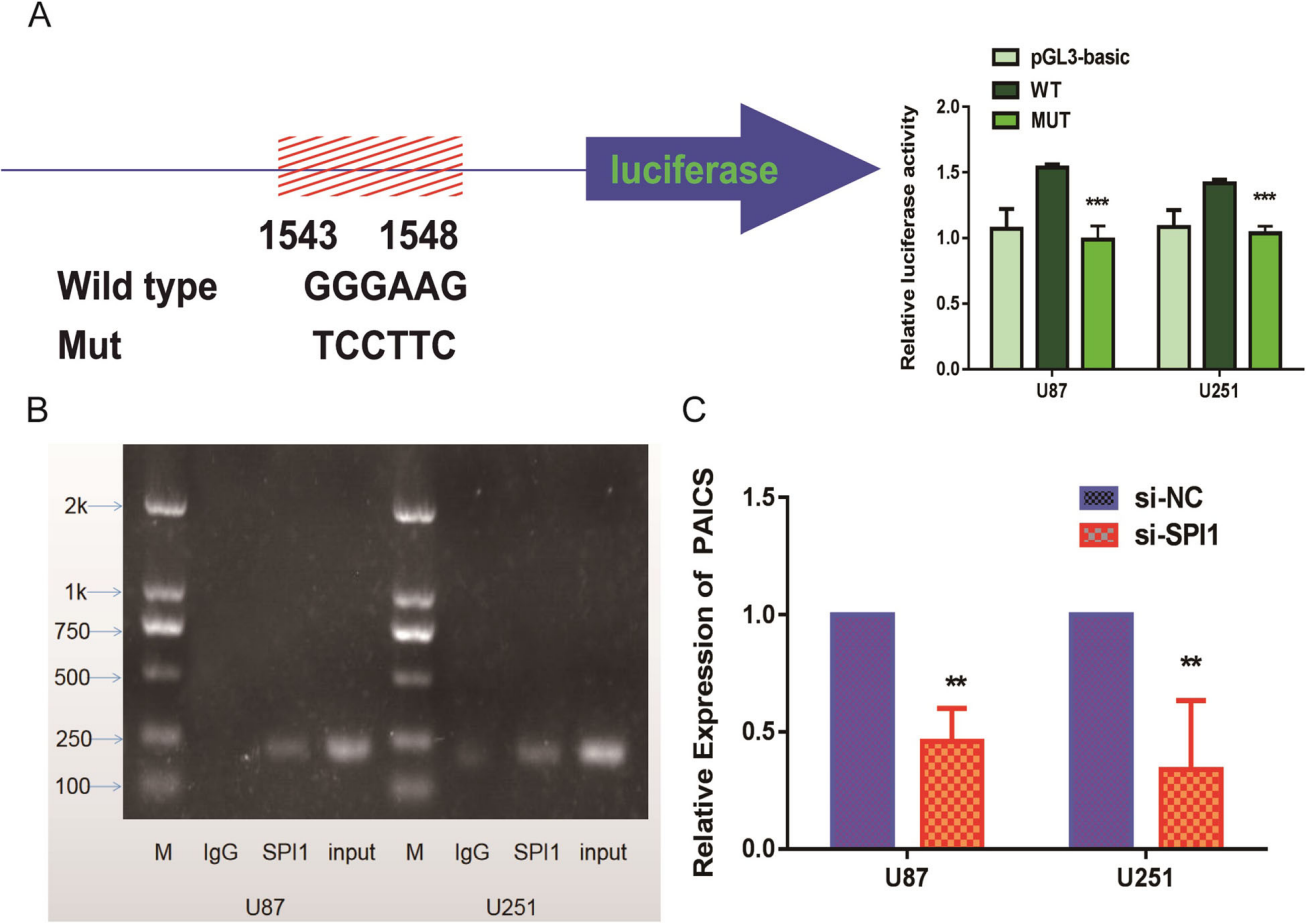
SPI1 binds to the promoter regions of PAICS and regulates PAICS expression. **a** Schematic of the PAICS promoter luciferase construct is depicted with the locations of the GGGAAG element and the sequences of mutation. Relative luciferase activities of the Wt or Mut GGGAAG regions in U87 and U251 cells were examined after transfecting with SPI1 48 hour later. The values of PGL3-WT and PGL3-Mut were relative to PGL3-basic. Data are mean ± SD for triplicate samples. ***, *p* < 0.001. **b** SPI1 binding at the promoter region of PAICS containing the GGGAAG element was assessed by CHIP assay. **c** PAICS RNA levels after transfecting the SPI1 interference fragment into the U87 and U251 cells. PAICS expression was normalized to GAPDH. Error bars indicate mean ±SD of 3 independent experiments. *, *p* < 0.05; ** *p* < 0.01; ***, *p* < 0.001

We analyzed the potential transcription factor binding sites in the promoter regions of PAICS from the online database JASPAR (http://jaspar.genereg.net/), hTFTARGET (https://bio.tools/hTFtarget) and HumanTFDB (http://bioinfo.life.hust.edu.cn/HumanTFDB#!/). We found that the promoter regions could be possible binding sites recognized by SPI1 (Supplementary Fig. S2) [15–17]. We observed that SPI1 effectively stimulated the luciferase activity of PAICS promoter in U87 and U251 cells (Fig. 4a). Moreover, ChIP results indicated that the transcription factor SPI1 can bind to the PAICS promoter region (Fig. 4b). Finally, we conducted knockdown of SPI1, which led to a decrease in the expression of PAICS in U87 and U251 cells (Fig. 4c). These results indicate that SPI1 can directly bind to the promoter region and regulate the expression of PAICS.

### SPI1 promotes glioma cell proliferation and migration through PAICS

**Fig. 5.**
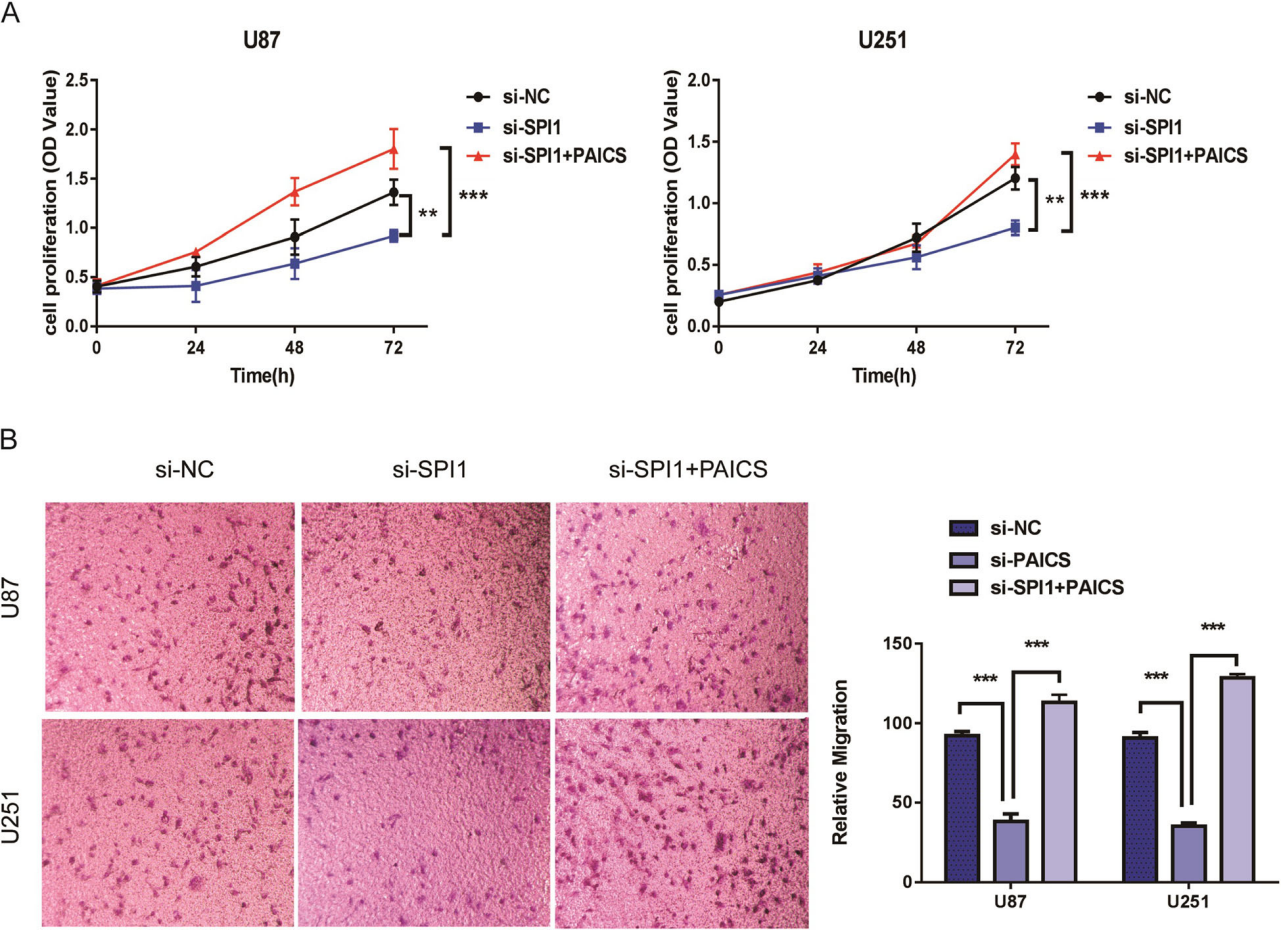
SPI1 promotes glioma cell proliferation and migration through PAICS. **a** PAICS can reverse the reduction of cell proliferation induced by SPI1 interference on the basis of CCK8 assays. Error bars represent the mean ± SD of 3 independent experiments. **, *p*< 0.01;***, *p*< 0.001. **b** PAICS can reverse the reduction of cell migration induced by SPI1 interference on the basis of transwell assays. The bar chart represents the migration cell numbers. Error bars represent the mean ± SD of 3 different field. ***, *p*< 0.001

Next, we sought to evaluate whether SPI1 can regulate cell proliferation and migration through PAICS. We used Western blot to detect the protein expression of SPI1 and PAICS. Meanwhile, we conducted the CCK8 cell experiment and transwell assay to detect cell proliferation and migration. The results demonstrated that PAICS was able to reverse the decrease in proliferation and migration of glioma cells induced by SPI1 interference (Fig. 5a, b).

## Discussion

Glioma is one of the most common brain tumors that is associated with high mortality. The SPI1 transcription factor belongs to ETS (e-twenty six) transcription factor family [18, 19], which is one of the largest families of transcription factors, comprised of 28 genes [20]. All ETS family members have a highly conserved DNA binding domain, known as the ETS domain, which has a helix-to-helix flank that binds to DNA binding sites with the GGA(A/T) sequence [21, 22]. The ETS family has many functions that are related to the development of different tissues and cancer, including regulation of cell differentiation, cell cycle regulation, cell migration, cell proliferation, apoptosis, angiogenesis, and other functions [23, 24]. Many ETSs have been associated with cancer, such as ERG (ETS-Related Gene), through gene fusion. When ERG transcription factors fuse with the EWS Gene, it causes Ewing's sarcoma [25, 26]. As a member of the ETS family, SPI1 plays an important role in the development of bone marrow and B lymphocytes, as well as activation of corresponding gene expression in tumors [27]. SPI1 binds to a PU-box sequence near the promoter of target genes, a purine-rich DNA sequence, and coregulates its expression in concert with other transcription factors and cofactors. The SPI1 protein can also bind RNA in order to regulate selective splicing of target genes [28].

In breast cancer, lung cancer and cervical cancer, SPI1 plays a role in promoting cancer [29]. In glioma, results have shown that SPI1 plays a cancer-promoting role [30, 31], but the functional verification and mechanism of SPI1 have not yet been reported. In this study, we investigated expression, function and mechanism of SPI1 in glioma. Compared to the normal group, the SPI1 mRNA and protein are up-regulated in glioma tissues. Moreover, we discovered that suppression of SPI1 decreases glioma cell growth and migration. Therefore, the results demonstrated that SPI1 exerts an oncogenic effect to promote cell growth and migration in glioma.

The mechanism of how SPI1 promotes glioma progression remains uncertain. One study has reported that PAICS can promote tumor proliferation and migration in neuroblastoma [32], though it has also been shown to play an important role in a variety of tumors [4–6]. Our previous study also found that PAICS plays an oncogene role in glioma [9]. Bioinformatics analysis indicated that SPI1 can bind to the PAICS promoter region. We speculate that SPI1 can play a role in promoting cancer through PAICS. Experimental results indicated that SPI1 can regulate expression of PAICS, and dual luciferase reporter assay verified binding of SPI1 with the PAICS promoter region. Through the rescue experiment, we also validated that SPI1 can promote proliferation and migration of tumor cells in glioma through PAICS.

## Conclusion

In conclusion, our results indicate that SPI1 is a tumor promoting gene. When inhibited, it may inhibit proliferation and migration of glioma cells. The role of SPI1 in the development of glioma is related to regulation of PAICS. Our results indicate that SPI1 may be a promising target for the treatment of glioma.

## Supplementary Information

**Additional file 1: Figure S1.** Expression levels of SPI1 in glioblastoma tissues were predited by online database HPA. Use the Human Protein Atlas (HPA) online database to analyze the protein expression of SPI1. Above is the IHC results of normal brain tissues. Below is IHC results of glioma tissues.

**Additional file 2: Figure S2.** Prediction of the potential transcript factor binding sites in the promoter regions of PAICS from online database JASPAR, hTFTARGET and HumanTFDB. **a** Prediction of transcription factor binding site of JASPAR database. **b** Prediction of transcription factor binding site of hTFTARGET database. **c** Prediction of transcription factor binding site of HumanTFDB database.

## Data Availability

All data relevant to the study are included in the article. The datasets used or analyzed during the current study are available.

## Abbreviations

GBM: Glioblastoma
ETS: E-twenty six
BBB: Blood-brain barrier
ERG: ETS Related Gene
PAICS: Phosphoribosylaminoimidazole Carboxylase And Phosphoribosylaminoimidazolesuccinocarboxamide Synthase
PLB: Passive Lyrsis Buffer
ChIP: Chromatin immunoprecipitation
FBS: Fetal Bovine Serum
