# Supplementary figures and images for: Study on the role of transcription factor SPI1 in the development of glioma
Source: Chin Neurosurg J. 2022 Apr 1;8:7. doi: 10.1186/s41016-022-00276-2 (PMC8973577; doi:10.1186/s41016-022-00276-2)

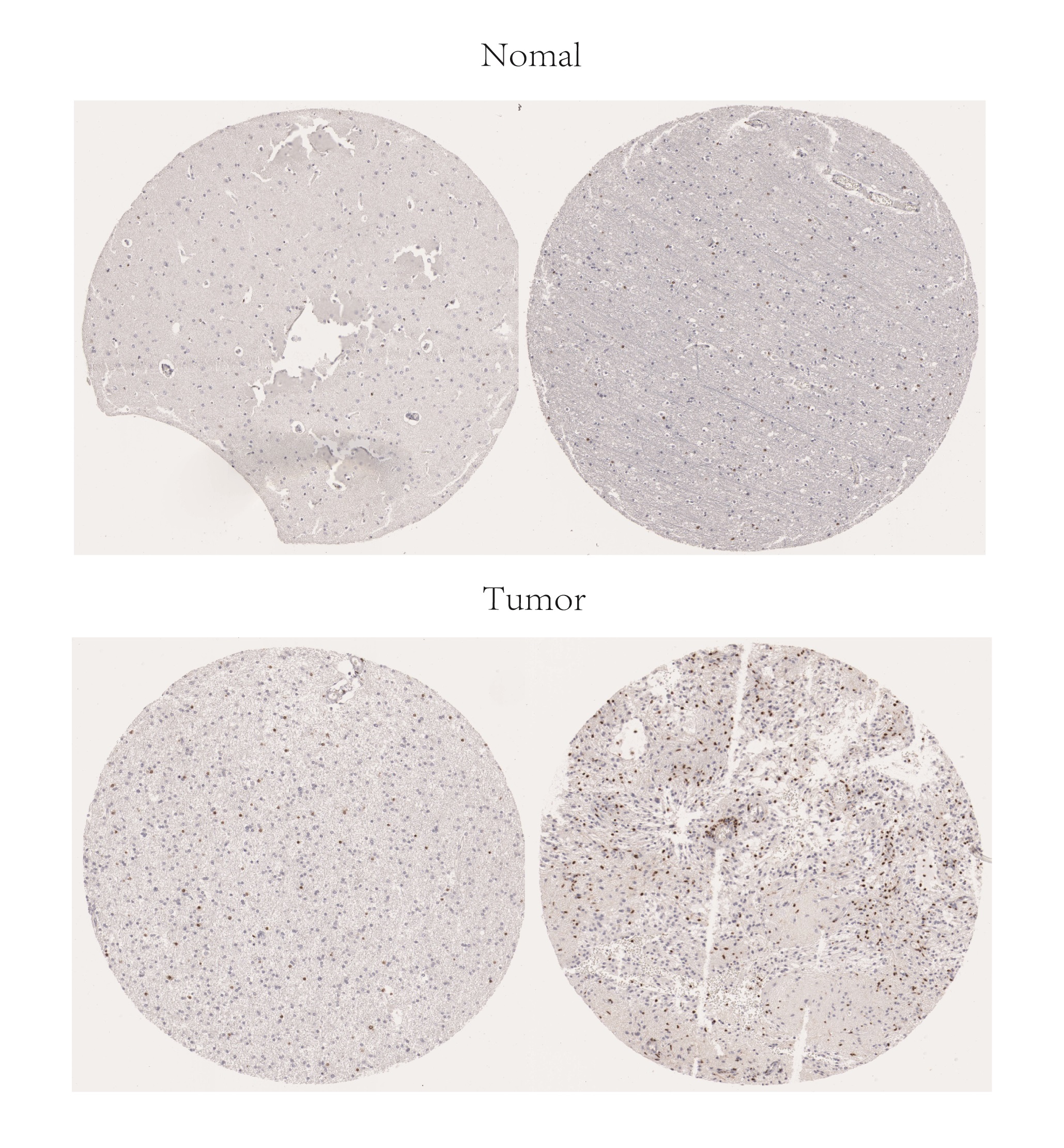

Supplement: Supplementary file 1 — Additional file 1: Figure S1. Expression levels of SPI1 in glioblastoma tissues were predited by online database HPA. Use the Human Protein Atlas (HPA) online database to analyze the protein expression of SPI1. Above is the IHC results of normal brain tissues. Below is IHC results of glioma tissues. [file 41016_2022_276_MOESM1_ESM.jpg]

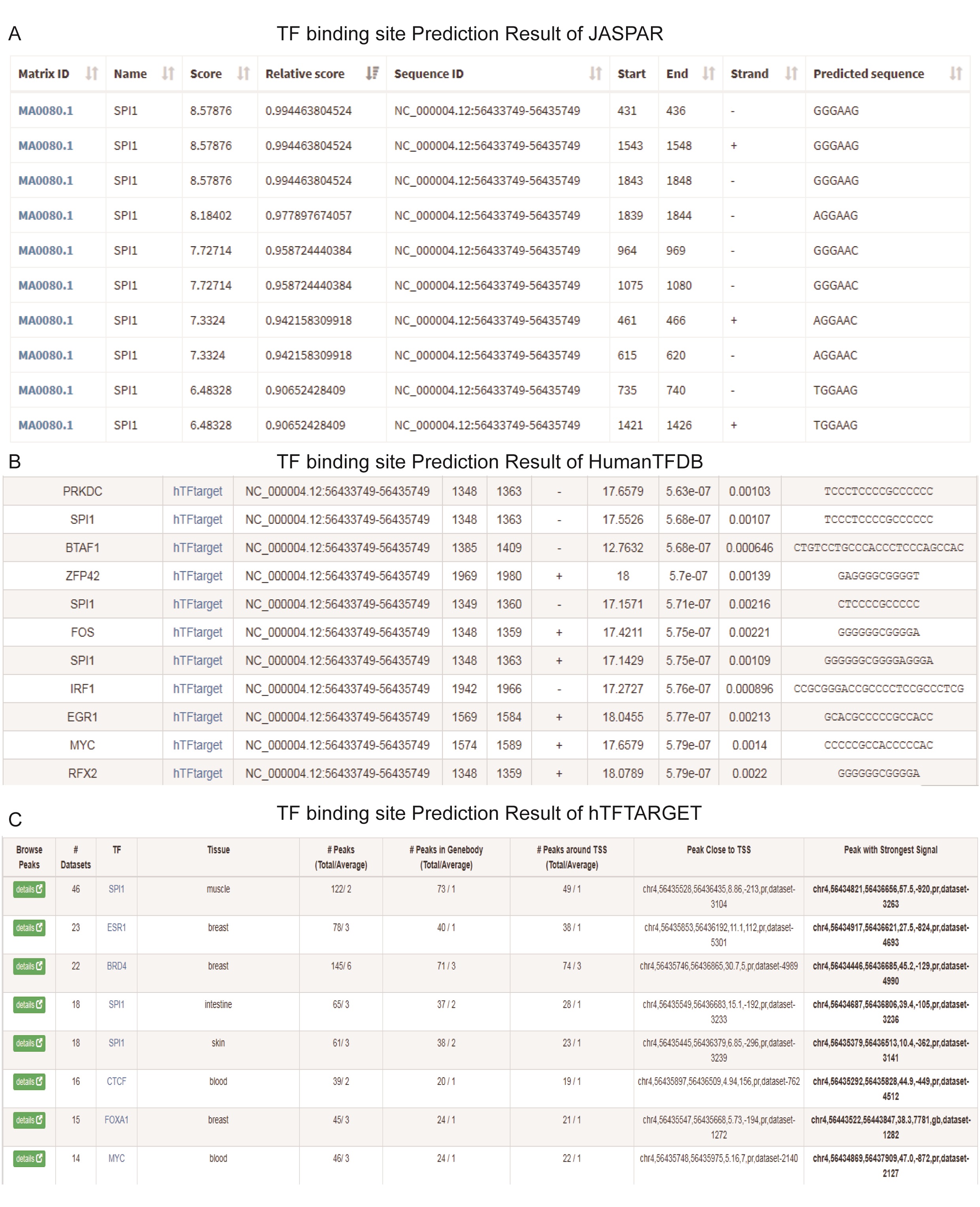

Supplement: Supplementary file 2 — Additional file 2: Figure S2. Prediction of the potential transcript factor binding sites in the promoter regions of PAICS from online database JASPAR, hTFTARGET and HumanTFDB. a Prediction of transcription factor binding site of JASPAR database. b Prediction of transcription factor binding site of hTFTARGET database. c Prediction of transcription factor binding site of HumanTFDB database. [file 41016_2022_276_MOESM2_ESM.jpg]
